# Risk of primary osteoporosis score (RPOPs): an algorithm model for primary osteoporosis risk assessment in grass-roots hospital

**DOI:** 10.1186/s12891-022-06014-0

**Published:** 2022-12-01

**Authors:** Xinhua Jiang, Na Yan, Yaqin Zheng, Jintao Yang, Yanfei Zhao

**Affiliations:** 1 Department of Laboratory Medicine, Zhejiang Quhua Hospital, Quzhou, Zhejiang Province China; 2 Key Laboratory of Digital Technology in Medical Diagnostics of Zhejiang Province, Dian Diagnostics Group Co.,Ltd, Hangzhou, Zhejiang Province China; 3 Department of Laboratory Medicine, Quzhou Maternal and Child Health Care Hospital, Quzhou, Zhejiang Province China

**Keywords:** Primary osteoporosis, Disease screening, miRNA, β-CTX, t-P1NP, LASSO, Hazard Assessment

## Abstract

**Background:**

This study aimed to develop and validate a lasso regression algorithm model which was established by correlation factors of bone mineral density (BMD) and could be accurately predicted a high-risk population of primary osteoporosis (POP). It provides a rapid, economical and acceptable early screening method for osteoporosis in grass-roots hospitals.

**Methods:**

We collected 120 subjects from primary osteoporosis screening population in Zhejiang Quhua Hospital between May 2021 and November 2021 who were divided into three groups (normal, osteopenia and osteoporosis) according to the BMD T-score. The levels of three micro-RNAs in the plasma of these people were detected and assessed by qRT-PCR. At the same time, the levels of β-CTX and t-P1NP in serum of the three groups were determined. Based on the cluster random sampling method, 84 subjects (84/120, 70%) were selected as the training set and the rest were the test set. Lasso regression was used to screen characteristic variables and establish an algorithm model to evaluate the population at high risk of POP which was evaluated and tested in an independent test cohort. The feature variable screening process was used 10-fold cross validation to find the optimal lambda.

**Results:**

The osteoporosis risk score was established in the training set: Risk of primary osteoporosis score (RPOPs) = -0.1497785 + 2.52Age − 0.19miR21 + 0.35miR182 + 0.17β-CTx. The sensitivity, precision and accuracy of RPOPs in an independent test cohort were 79.17%, 82.61% and 75%, respectively. The AUC in the test set was 0.80. Some risk factors have a significant impact on the abnormal bone mass of the subjects. These risk factors were female (*p* = 0.00013), older than 55 (*p* < 2.2e-16) and BMI < 24 (*p* = 0.0091) who should pay more attention to their bone health.

**Conclusion:**

In this study, we successfully constructed and validated an early screening model of osteoporosis that is able to recognize people at high risk for developing osteoporosis and remind them to take preventive measures. But it is necessary to conduct further external and prospective validation research in large sample size for RPOPs prediction models.

**Supplementary Information:**

The online version contains supplementary material available at 10.1186/s12891-022-06014-0.

## Background

Primary osteoporosis (POP) is the most common bone disease which characterized by low bone mass, damage to bone microstructure, increased bone fragility and prone to fracture [1]. POP affect family burden and quality of life through associated fragility fractures [2]. There are nearly 9 million fragility fractures caused by osteoporosis every year worldwide [3]. These patients are prone to disability, with a low independence in activities of daily living and a poor quality of life [4–6]. Although effective drug prevention measures and rehabilitation treatment measures have been established for fragile fractures, the effects are inconsistent [7–9]. Early detection and early intervention are very important to improve the life quality of the Osteoporosis patients [10–12]. It is one of the public health issues which we are most concerned [13]. The main ways to reduce its endangerment are early detection, early intervention and early treatment. Therefore, population screening is very important for the prevention of the disease [14, 15].

Dual Energy X-ray Absorptiometry (DXA) is the gold standard for the diagnosis of osteoporosis [16, 17]. However, due to the lack of portability of measuring instruments, high inspection cost, long time-consuming detection, and the insufficient cognitive level of grass-roots medical staff on the basis of osteoporosis diagnosis, it is not suitable for population screening [15]. At present, some simple osteoporosis screening tools are mostly used to help doctors screen patients for the first time [18]. After reaching the tool score threshold, it is recommended to use DXA to detect bone mineral density [18–20]. For example, International Osteoporosis Foundation (IOF), Fracture Risk Assessment Tool (FRAX), osteoporosis self-assessment tool for Asians (OSTA). However, the screening effect of these tools is not ideal. Because there are few variables and the influencing factors of primary osteoporosis are not considered. The prediction results of IOF one minute osteoporosis risk test come from the questionnaire survey of subjects. It contains 19 questions, some of which involve the subjects’ privacy, such as whether they have had impotence, decreased libido, etc. In these questions, as long as the answer to one question is “yes”, it will be positive, indicating that there is a risk of osteoporosis and bone density examination is recommended [20]. The questionnaire survey are easily affected by the subjective cognition of the subjects, so that the validity and authenticity of survey results cannot be effectively guaranteed. Akram Kharroubi evaluated the effectiveness of IOF in postmenopausal Palestinian women by Receiver operating characteristic (ROC) that was only 0.629 [21]. Fracture-related risk factors in FRAX were identified from a global large sample meta-analysis. In practical application, FRAX needs epidemiological data of fracture incidence and mortality in corresponding countries. Therefore, FRAX thresholds are constantly adjusted and optimized. Currently, there are 86 versions of FRAX models [22, 23]. OSTA only considered two variables: OSTA index = [weight (kg) - age(years)]*0.2. OSTA is only applicable to postmenopausal women, and its specificity is not very well, which needs to be judged in combination with other risk factors [24]. One study showed that OSTA has a sensitivity of 79% and specificity of 60% in screening in 722 southern Chinese postmenopausal women [24]. Therefore, it is of great significance to establish a rapid, economical and accurate early screening method for primary osteoporosis.

Bone turnover is a renewal and replacement process of old bone absorption and new bone formation in bone tissue [25, 26]. The key pathophysiological mechanism of many bone diseases is bone turnover imbalance [27, 28]. In normal people with different ages and different disease states, the dynamic state of systemic bone metabolism can be reflected by the changes of bone turnover biomarkers (BTMs) in blood or urine [29, 30]. The most commonly used BTMs in clinical research are bone resorption marker β isomer of C-terminal telopeptide of type I collagen (β-CTX) and bone formation marker total type I collagen amino terminal lengthening peptide (t-P1NP) that are also recommended indicators by International Osteoporosis Foundation (IOF) [31, 32].

MicroRNAs (miRNAs) are non-coding single-stranded small RNAs which is involved in the regulation of post-transcriptional gene expression [33, 34]. Studies on bone conversion have found that abnormal expression of miRNAs can affect bone resorption, bone formation and osteoporosis [35, 36]. More and more studies have focused on the diagnostic value of miRNAs in osteoporosis [37, 38]. Among the members of the miRNA family, miR-182 is located on human chromosome 7 (7q32.2), and through the negative regulation of different target mRNAs, it participates in various physiological and pathological processes of biology and exerts different functions [39]. Inoue Kazuki found that the expression level of miR-182 was significantly different in different tissues, and was highly expressed in cortical bone, metaphysis, and adipose tissue, suggesting that miR-182 may be closely related to the physiological functions of bone and adipocytes [39]. Li Hong-qiu found that miR-21 and miR-133a are sensitive serum markers for postmenopausal osteoporosis in women [40]. Wu Xiao-Hui and Wang Gang also found that miR-21 and miR-133a caused dysfunction of BMSCs in osteoporosis [41].

To sum up, this study aimed to develop and validate a early osteoporosis screening model which was established by correlation factors of bone mineral density and could be predicted a high-risk population of POP.

## Methods

### Patients and samples:

A total of 120 people from primary osteoporosis screening population were collected which were enrolled from Zhejiang Quhua Hospital between May 2021 and November 2021. The inclusion criteria were as follows: (1) bone status data measured by quantitative ultrasound (QUS) bone densitometry; (2) miRNA-21, miRNA-133a and miRNA-182 level andβ-CTX, t-P1NP level measured by plasma tests. The exclusion criteria were as follows: (1) bone problems or suffered from bone related health complications have been previously diagnosed; (2) the subjects took any prescription drugs or food supplements that might affect bone condition; (3) the subjects without plasma samples; (4) the subjects with failed repeated detection of plasma samples.All recruited subjects were divided into three groups (normal, osteopenia and osteoporosis) according to the BMD T-score. A T-score of ≥ − 1.0 was classified as normal; a T-score between > − 2.5 and < − 1.0 was classified as osteopenia; and a T-score of ≤ − 2.5 was classified as osteoporosis (Supplementary Table S1).

Two tubes of fasting blood samples were obtained, 2ml for each tube. All the separated plasma samples and serum samples were stored in liquid nitrogen. Serum samples were used to measure the levels of bone conversion markers β-CTX and t-P1NP. Plasma samples were used to detect the relative expression levels of miRNA-21, miRNA-133a and miRNA-182. This study was approved by the Human Research Ethics Committee of Zhejiang Xinhua Hospital (No.2019SJGY03) and written informed consent was obtained from all participants before study commencement.

### Detection of bone conversion markers β-CTX and t-P1NP:

Serum samples β-CTX and t-P1NP levels were detected by electro chemiluminescence immunoassay (ECLl). The Roche Cobas E602 Automatic immuno-analyzer (Roche, Rotkreuz, Switzerland) was used for detection. Serum samples were measured for each time, two levels of quality control materials were measured at the same time, and the results were within the specified allowable range (Supplementary Table S1).

### Detection of relative expression levels of miRNA-21, miRNA-133a and miRNA-182:

The total RNA from plasma samples was extracted by a BIOG cfRNA Easy Kit (BIOG, ChangZhou, China) following the manufacturer’s instructions. The reverse transcription experiment was carried out by Goldenstar™ RT6 cDNA Synthesis Kit Ver2 (Tsingke, Beijing, China). The cDNA product was diluted as a qPCR template and amplified with 2×T5 Fast qPCR Mix(SYBR Green I, Tsingke, Beijing, China).

A Real-time PCR System (BIOER) was used to amplify. The amplification curves were analyzed by the determination of Cp (by the second derivative method), as well as for a melting curve analysis. U6 RNA was used as endogenous control in all experimental reactions. The relative expression levels of each miRNA was determined by 2^–ΔΔCT^ method (Supplementary Table S1). Primer sequences of miRNA-21, miRNA-133a and miRNA-182 were shown in supplementary material (Supplementary Table S2).

### Derivation process of algorithm model

**Fig. 1:**
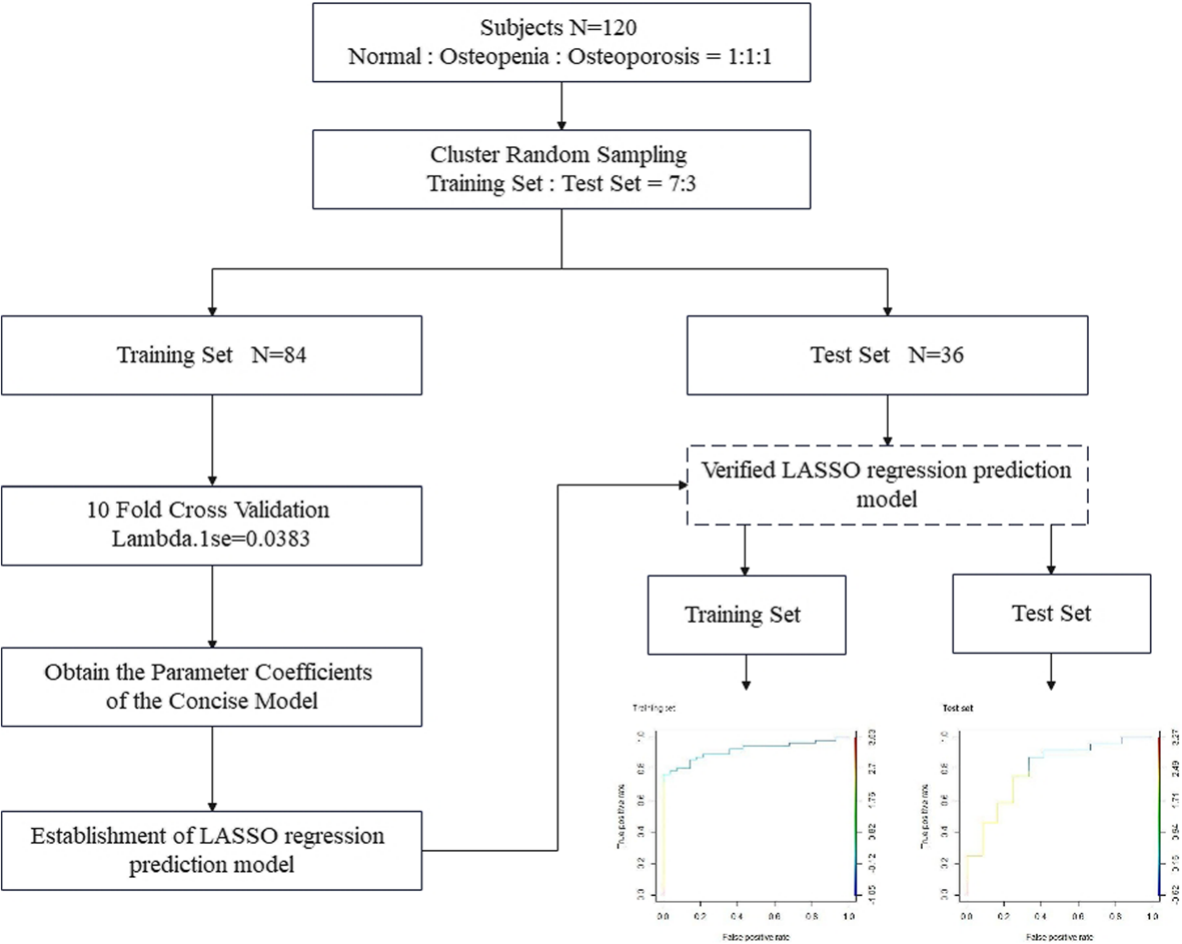
Schematic for development of RPOPs. 120 subjects were split into training and independent test sets by the cluster random sampling method. The feature variable screening process was used 10 fold cross validation in training set. Lasso regression was used to screen characteristic variables and establish a algorithm model to evaluate the population at high risk of POP which was evaluated and tested in an independent test cohort

The classification machine learning algorithm was used to predict the high-risk population of osteoporosis, which was encoded as binary result variables (normal vs. osteopenia/osteoporosis). Based on the cluster random sampling method, 84 subjects(84/120, 70%) were selected as the training set and the rest were the test set (Fig. 1 and Supplementary Table S3). All test results shall be subject to Z-score standardization (Supplementary Table S3) and category variable assignment is used for subsequent algorithm analysis (Supplementary Table S4). Lasso regression was used to screen characteristic variables and establish an algorithm model to evaluate the population at high risk of POP in the training cohort which was evaluated and tested in an independent test cohort. The feature variable screening process was used 10 fold cross validation to find the optimal lambda: lambda.1se = 0.0383. A concise model with 4 parameters was acquired: Risk of primary osteoporosis score (RPOPs) = -0.1497785 + 2.52Age − 0.19miR21 + 0.35miR182 + 0.17β_CTx. The AUC in the training set was 0.92 and 0.80 in the test set (Supplementary Figure S1). At the same time, we also tested the prediction results of OSTA.

### Statistical analysis

R programming language was used for all analyses. R version 4.1.1 (2021-08-10) was used for the classification and training of the prediction model (Fig. 1). Kruskal Wallis test was used to analyze the significant differences among groups. All tests were 2-sided, with *p* ≤ 0.05 as the criterion standard for determining signifificance.

## Results

### A lasso regression model to predict the performance of high-risk population in primary osteoporosis

In the training set, we acquired a concise model with 4 parameters: Risk of primary osteoporosis score (RPOPs) = -0.1497785 + 2.52Age − 0.19miR21 + 0.35miR182 + 0.17β_CTx. Forty-seven (55.95%) subjects were predicted as high-risk population of primary osteoporosis by RPOPs, and thirty-seven (44.05%) subjects were low-risk population. Among the forty-seven high-risk populations, forty-five (95.74%) were identified as abnormal bone mass (osteopenia or osteoporosis) by DXA. Among the thirty-seven low-risk populations, twenty-six (70.27%)were determined to normal bone mass by DXA. The AUC in the training set was 0.92. Therefore, we concluded that patients with high RPOPs score (RPOPs = 1) had abnormal bone mass. This kind of population is more likely to develop primary osteoporosis. Therefore, we concluded that patients with high RPOPs score (RPOPs = 1) had abnormal bone mass. This kind of population is more likely to develop primary osteoporosis, while those with low RPOPs scores had normal bone mass.

**Fig. 2:**
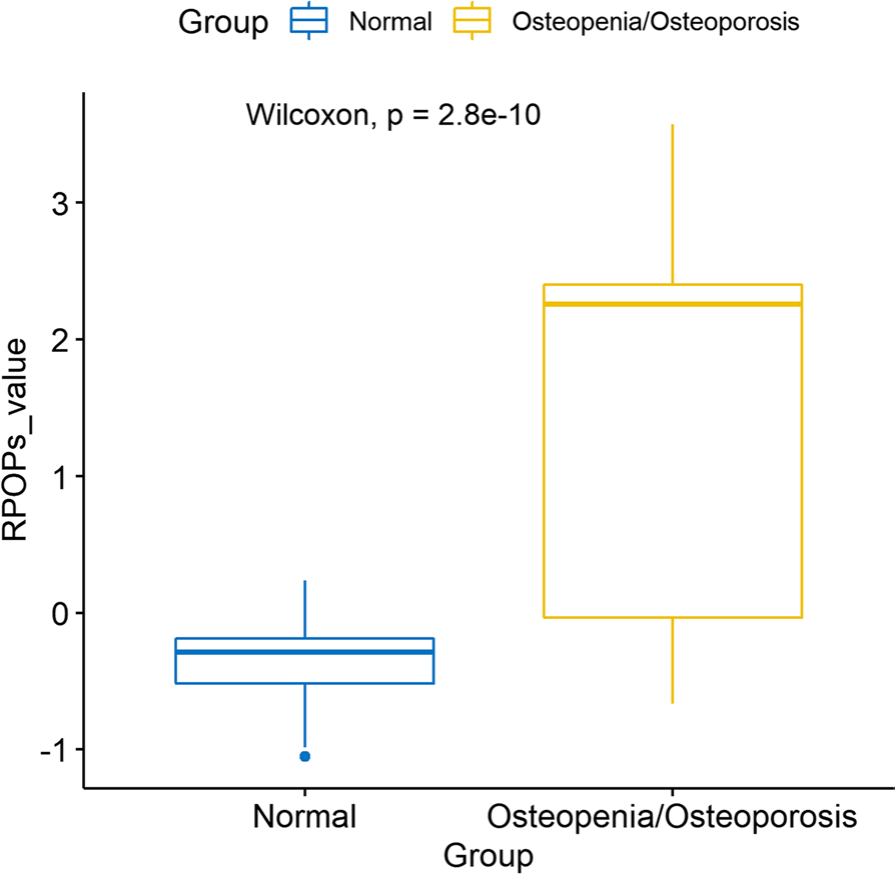
RPOPs predicted primary osteoporosis high-risk population. Box-whisker plots of RPOPs values. RPOPs, risk of primary osteoporosis score

In the validation cohort, we evaluated the prediction performance of RPOPs and verified our hypothesis. Among the thirty-six subjects in the validation set, twenty-three (63.89%)subjects were predicted by RPOPs to be at high risk for primary osteoporosis, of which nineteen (82.61%) subjects were classified as abnormal bone mass (osteopenia / osteoporosis) by DXA. The AUC in the test set was 0.80. We verified that the sensitivity, precision and accuracy of RPOPs were 79.17%, 82.61% and 75%, respectively (Table 1). In addition, our hypothesis was validated: subjects with high RPOPs score suggested a high risk of primary osteoporosis. In contrast, subjects with lower RPOPs score had normal bone mass (Fig. 2).

**Table 1:** Prediction of RPOPs in primary osteoporosis high-risk population

	RPOPs = 1	RPOPs = 0
Training cohort (*N* = 84)	47	37
Abnormal bone mass	45 (45/47, 95.74%)	11 (11/37, 29.73%)
Bone mass normal	2 (2/47, 4.26%)	26 (26/37, 70.27%)
Test cohort (*N* = 36)	23	13
Recurrence and metastasis	19 (19/23, 82.61%)	5 (5/13, 38.46% )
Disease progression-free	4 (4/23, 17.39%)	8 (8/13, 61.54%)
Sensitivity	79.17%	
Accuracy	82.61%	
Precision	75.00%	

### Comparison of the predictive effectiveness

OSTA is the most commonly used screening tool for osteoporosis in primary hospitals. The diagnosis and treatment guidelines for primary osteoporosis recommend that people with OSTA<-1 need DXA test to further determine the bone mineral density level. However, the limitation of OSTA is that it is only applicable to postmenopausal women.

We selected 42 postmenopausal women from 120 subjects in this study which included three subjects with normal bone mass and 39 subjects with abnormal bone mass. The OSTA score was used to assess the risk of primary osteoporosis in these subjects. The results showed that in 42 postmenopausal women, the sensitivity, accuracy and precision of OSTA score were 82.05%, 78.57% and 94.12% respectively (Table 2). The predictive sensitivity of RPOPs in 42 postmenopausal women reached 100%, accuracy reached 92.86% and precision reached 95.12%. The comparison results of the two methods show that RPOPs had better sensitivity, accuracy and precision than OSTA. The performance of accuracy is outstanding: RPOPs 92.86% vs. OSTA 82.61%.

**Table 2:** Comparison of RPOPs and OSTA prediction results in postmenopausal women

Postmenopausal women *N* = 42	RPOPs = 1	RPOPs = 0	OSTA<-1	OSTA≧-1
Forecast results	41	1	34	8
Abnormal bone mass	39 (39/41, 95.12%)	0 (0/1, 0.00%)	32 (32/34, 94.12%)	7 (7/8, 87.50%)
Bone mass normal	2 (2/41, 4.88% )	1 (1/1, 100.00%)	2 (2/34, 5.88%)	1 (1/8, 12.50%)
Sensitivity	100.00%		82.05%	
Accuracy	92.86%		78.57%	
Precision	95%		94%	

### Major risk factors for primary osteoporosis

According to the predicted value of RPOPs, the effect of general indicators on bone mass was observed. The results of data analysis showed that women were more likely to obtain high RPOPs score (*p* = 0.00013). The abnormal bone mass was more serious, when the subjects were older than 55 (*p* < 2.2e-16). Subjects with a BMI < 24 need to pay more attention to their bone health (*p* = 0.0091).(Supplementary Figure S2).

## Discussion

With the continuous development of the study on the pathological mechanism of osteoporosis, it is found that the basic pathological mechanism is the defect of the coupling between bone absorption and bone formation in the process of bone metabolism. It leads to the imbalance of calcium and phosphorus metabolism in the human body and the gradual reduction of bone mineral density [27, 28]. However, with the development of bone transformation research, the abnormal expression of miRNAs is considered to affect bone resorption and bone formation [35, 36]. Bone metabolism markers and miRNAs have not been covered by early screening algorithm models for osteoporosis. In this study, based on the markers of bone turnover and miRNAs, combined with general clinical data, we established an algorithm model to evaluate the high-risk population of primary osteoporosis.

Our study confirmed the previous findings that women, old age and low BMI may be the predictors of osteoporosis. In addition, this study confirms the value of miRNAs biomarkers in assessing bone metabolic abnormalities. Importantly, for the first time, we used the heterogeneous predictors, including subjects’ general information (gender, age, BMI), markers of bone turnover (β-CTX and t-P1NP) and miRNAs, a new prediction model RPOPs was constructed by lasso regression to distinguish the population with normal bone mass and abnormal bone mass. This reduced model includes four predictors: Age, miR21, miR182 and β-CTx. Compared with other early screening models for osteoporosis, these predictive factors can objectively evaluate the bone mass and exclude the influence of subjective factors on the prediction results. Since the outbreak of COVID-19 in China, grass-roots hospitals have been required to have nucleic acid testing conditions. In order to meet this requirement, primary hospitals have purchased real-time PCR instruments. With the gradual stabilization of the COVID-19 epidemic, how to efficiently use these idle instruments is a problem for managers. The miRNA detection in this study can promote the planning of saturated use of real-time PCR instruments in grassroots hospitals. It is also a basic condition for grass-roots hospitals to carry out miRNA detection. In the future, RPOPs will help grass-roots hospitals to identify high-risk groups of osteoporosis and prevent the harm caused by osteoporosis in advance.

Unfortunately, there were limitations in this study. First of all, the bone mass of the subjects in this study was determined after DXA test, and the blood samples were collected to detect the molecular indicators. The questionnaire was not designed, so the pregnancy and childbirth status of female subjects was not clear. It was impossible to assess whether smoking, drinking and other lifestyle habits have an impact on bone mass. Due to limited information, only OSTA predictive performance was compared. Secondly, the size of data sets has some limitations, but they perform well in the current model tests. It is necessary to carry out external and prospective validation research on RPOPs prediction model. Thirdly, detection and analysis of miRNA and β-CTx needs to be completed by professional experimental technicians, which may increase labor and testing costs. This requirement may lead to the limitation of RPOPs in practical application. Finally, in the model design, a binary classification model was established, which was limited by the sample size. In this study, a multi-classification model can be attempted to make a more detailed distinction among the subjects. However, considering that people with osteopenia needed to consider treatment as appropriate and were mainly used for screening in grass-roots hospitals, people with osteoporosis and osteopenia were regarded as risk groups of osteoporosis.

In conclusion, we have for the first time developed a simplified predictive model by integrating the biomarkers of bone turnover, miRNAs and subject characteristics that can objectively evaluate high-risk groups of osteoporosis in grass-roots hospitals.

## Supplementary Information

**Additional file 1: Table S1. **Patients’ clinicalinformation, the relative expression levels of miRNAs in plasma and the levelsof bone conversion markers β-CTX and T-P1NP in serum. **Table S2.** Primer sequences (5'to3'). **Table S3.** All the patients’ clinical information and the detection of relative expression levels were assigned a value. **Table S4.** Clinical variables assignment.

**Additional file 2: Figure S1. **Receiver operating characteristic curve (ROC) oftraining set and test set. The ordinate means sensitivity.The abscissa means the difference between 1 and specificity. **Figure S2. **RPOPs associateswith general characteristics.Box-whisker plots of RPOPsvalues for general characteristics.

## Data Availability

The datasets used or analysed during the current study are available from the corresponding author on reasonable request.

## Abbreviations

POP: Primary Osteoporosis
DXA: Dual energy X-ray absorptiometry
BMD: Bone Mineral Density
BTMs: Bone Turnover Biomarkers
β-CTX: βisomer of C-terminal telopeptide of type I collagen
t-P1NP: Total type I collagen amino terminal lengthening peptide
IOF: International Osteoporosis Foundation
BMSCs: Bone marrow mesenchymal stem cells
BMI: Body Mass Index
ROC: Receiver operating characteristic curve
AUC: Area Under Curve
SD: Standard Deviation
RPOPs: Risk of primary osteoporosis score
