# Supplementary material for: Risk of primary osteoporosis score (RPOPs): an algorithm model for primary osteoporosis risk assessment in grass-roots hospital
Source: BMC Musculoskelet Disord. 2022 Dec 1;23:1041. doi: 10.1186/s12891-022-06014-0 (PMC9713074; doi:10.1186/s12891-022-06014-0)

Risk of primary osteoporosis score(RPOPs): An algorithm model for primary osteoporosis risk assessment in grass-roots hospital

**SUPPLEMENTARY DATA（Figures can be published online.）**

**Figure S1. Receiver operating characteristic curve (ROC) of training set and test set.** The **o**rdinate means sensitivity. The abscissa means the difference between 1 and specificity.


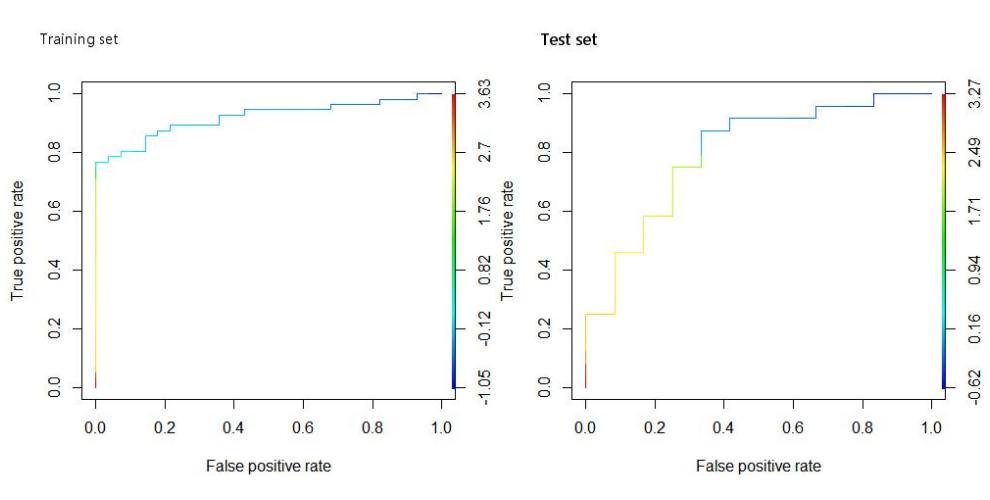


**Figure S2.** **RPOPs associates with** **general characteristics.** Box-whisker plots of RPOPs values for general characteristics.


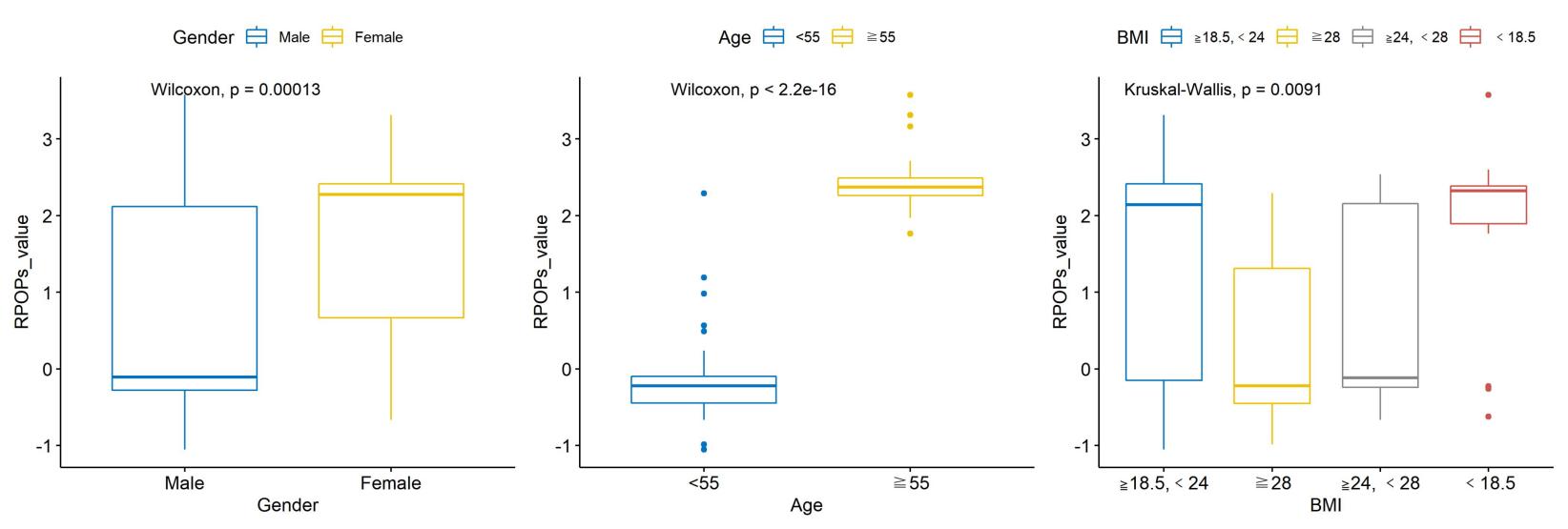

Supplement: Supplementary file 2 — Additional file 2: Figure S1. Receiver operating characteristic curve (ROC) oftraining set and test set. The ordinate means sensitivity.The abscissa means the difference between 1 and specificity. Figure S2. RPOPs associateswith general characteristics.Box-whisker plots of RPOPsvalues for general characteristics. [file 12891_2022_6014_MOESM2_ESM.docx]
